# Role of urodynamics in diagnosing tethered cord in spina bifida patients

**DOI:** 10.1186/1743-8454-7-S1-S5

**Published:** 2010-12-15

**Authors:** Pieter Dik, Sven Nadorp, Johan EH Pruijs, Rob HJM Gooskens, Tom PVM de Jong

**Affiliations:** 1 Wilhelmina Children’s Hospital, Lundlaan 6,Â 3584 EA Utrecht, POBOX 85090, The Netherlands

## Background

The aim of this study was to see how many patients with spina bifida aperta (SBA) developed a clinical significant tethered cord (TC) and how urodynamics influenced this diagnosis.

## Materials and methods

Between 2000 and 2008 approximately 400 SBA patients, under the age of 18 years, were followed. In 70 cases secondary TC was suspected, based on a variety of symptoms: visible back anomalies, loss of strength, changes in lower urinary tract function, increase in scoliosis or a combination of these. Patients had a one day check-up by a multidisciplinary team, consisting of neurologist, orthopedic surgeon, neurosurgeon, physical therapist and urologist. Imaging (MRI) and urodynamic studies were done on the same day. At the end of the day the results were evaluated and a decision was made whether or not to operate for TC.

## Results

26 patients with SBA had untethering or a myelotomy. 18 patients had neurologic changes. 7 of these patients also had changes in urodynamics. None of the 26 patients had urodynamic changes without neurologic deterioration. This is probably because all spina bifida patients were on oxybutynin and CIC. Ten patients had scoliosis and were treated by myelotomy to cure tethering and prevent Chiari complications.

## Conclusions

In a population of 400 SBA patients, 70 patients were seen in a single day check-up for TC suspicion. 26 patients (7%) were treated for symptomatic TC in 8 years time. In this study urodynamics did not contribute to the diagnosis, probably due to the use of antimuscarinics.

